# The intersectional impact of climate change and gender inequalities in Africa

**DOI:** 10.1002/puh2.169

**Published:** 2024-03-22

**Authors:** Olalekan John Okesanya, Khlood Fathi Hassan Alnaeem, Hakeem Kayode Hassan, Adebimpe Tolutope Oso, Olaniyi Abideen Adigun, Oumnia Bouaddi, Noah Olabode Olaleke, Sara Gabrallah M kheir, Usman Abubakar Haruna, Deborah Oluwaseun Shomuyiwa, Emery Manirambona, Melat Tesfaye Asebot

**Affiliations:** ^1^ Department of Medical Laboratory Science Neuropsychiatric Hospital, Aro Abeokuta Nigeria; ^2^ Community Medicine primary Health Care, Federal Ministry of Health Khartoum Sudan; ^3^ Department of Medical Laboratory Science Federal Medical Centre Bida Niger Nigeria; ^4^ Department of Medical Laboratory Science University College Hospital Ibadan Oyo Nigeria; ^5^ International School of Public Health Mohammed VI University of Health Sciences Casablanca Morocco; ^6^ Mohammed VI Center for Research and Innovation Rabat Morocco; ^7^ Obafemi Awolowo University Teaching Hospital Complex Ile‐Ife Osun Nigeria; ^8^ The Global Fund Program Management Unit, Federal Ministry of Health Khartoum Sudan; ^9^ Nazarbayev University School of Medicine Astana Kazakhstan; ^10^ Faculty of Pharmacy University of Lagos Lagos Nigeria; ^11^ College of Medicine and Health Sciences University of Rwanda Kigali Rwanda; ^12^ Global Health Focus Bujumbura Burundi; ^13^ Johns Hopkins Bloomberg School of Public Health Baltimore Maryland USA

**Keywords:** Africa, climate change, disasters, gender inequalities, women

## Abstract

The global pursuit of the United Nations Sustainable Development Goals underscores the importance of combating inequality, with climate change emerging as a significant threat, especially in low‐ and middle‐income countries (LMICs). This commentary explores the profound impact of climate change on the lives of women in Africa, shedding light on a critical issue where 80% of the female population in LMICs grapples with its far‐reaching consequences. Climate change is exacerbating existing gender disparities, particularly within the realms of agriculture, livelihoods, and resource access. Barriers like limited training and technology access hinder effective adaptation, perpetuating discrimination. Rooted deeply in social and cultural norms, the consequences of climate change negatively impact the human rights of women, resulting in increased vulnerability to illnesses, malnutrition, limited housing, and restricted support services. Health risks, including malaria transmission and respiratory diseases, further compound existing challenges, leading to increased rates of anemia, violence against women, alarming spikes in child marriages, and socio‐economic consequences. Integrated policies emphasizing gender mainstreaming, multisectoral approaches, and closing gender gaps in asset ownership are crucial to addressing these challenges. Education, training, and upskilling opportunities are essential to empowering women to confront climate change, further advocating for the development and enforcement of laws and policies recognizing gender differences and safeguarding women's rights. Moreover, there is a need for integrated solutions to foster sustainable development in Africa, as climate change is not a standalone issue but rather intertwines with various aspects of life. By advocating for policies that promote gender equality, education, and resource access, it seeks to pave the way for a more resilient and empowered female population, capable of navigating the complexities of climate change and contributing to the broader goal of sustainable development on the African continent.

## INTRODUCTION

Climate change, one of the challenges faced by humanity, has emerged as a predominant threat, impacting vulnerable populations worldwide, especially in low‐ and middle‐income countries [1, 2], where 70% of the 1.3 billion population are females, among whom a staggering 80% bear the brunt of climate change [3]. These impacts are significantly pronounced in Africa, a continent consistently identified as the most vulnerable to climate change.

Rooted in social and cultural norms, gender inequalities in Africa lead to divergent life expectancies, from 82 years in affluent nations to under 63 years in impoverished countries like Lesotho [4]. Despite Africa's economic vitality, women, responsible for 80% of food production and comprising over 60% of the agriculture workforce in Sub‐Saharan Africa, face heightened challenges due to climate‐induced crop changes, ranging from 48% in Burkina Faso to 73% in the DR Congo [5]. The pervasive effects of climate change exacerbate existing gender disparities, impeding women's growth potential. The global pursuit of the United Nations Sustainable Development Goals (SDGs) underscores the imperative to combat inequality and injustice on a global scale [1]. This demonstrates the importance of addressing gender gap linked to climate change in order to support involved decision makers with evidence. We carried out this study to delve into the intersection of climate change and gender inequalities, offering recommendations to address their impact on women.

## CLIMATE CHANGE AND AFRICAN WOMEN: A MULTIFACETED CHALLENGE

Climate change unfolds its challenges across regions and families relying predominantly on natural resources, particularly those with limited abilities to respond to short‐term natural crises. Famine, floods, drought, hurricanes, landslides, and the continuous deterioration of ecosystems present imminent threats, with women bearing high susceptibility [6]. The multifaceted impact on women in Africa extends beyond individual well‐being, encompassing agricultural productivity, livelihoods, gender roles, income, and resource access [7]. Effective adaptation becomes challenging for women due to barriers, such as limited access to training, extension services, and technology. Traditional gender roles further exacerbate these gender‐specific impacts [6].

Gender discrimination and disparities persist in Africa due to high rates of illiteracy, food shortages, poor health conditions, and the patriarchal system. Consequently, women face obstacles in addressing climate‐related issues like coastal erosion, flooding, and soil salinity, leading to civil, financial, and political limitations in rural settlements in developing countries [8]. The burden on women to fulfill essential tasks, such as childbearing, securing food, water, and energy for cooking, contributes to the perpetuation of gender inequalities. A study across 24 Sub‐Saharan African nations revealed that between 46% and 90% of adult females were the primary water collectors, a task consuming over 30 min of their time [9]. Additionally, 62% of female children in all 24 countries studied were responsible for gathering water, compared to 38% of male children [9, 10]. These responsibilities, coupled with the demands of consistent farming, resource gathering, and agribusiness, leave women with limited time for knowledge‐seeking, learning, skill acquisition, and income generation. Consequently, the higher illiteracy rate among females compared to males is perpetuated [10].

## IMPLICATIONS ON WOMEN

The consequences of climate change reverberate in profound ways, negatively impacting the human rights of numerous women in Africa. This manifests as increased hunger, susceptibility to illnesses, limited housing, restricted access to support services, loss of employment, and deprivation of financial and civil rights. These effects disproportionately affect the natural, physical, public, and economic assets of young girls and women [11]. Women's health in Africa is significantly affected by climate change, leading to malnutrition, chronic diseases, and reduced water quality, with heightened vulnerabilities during pregnancy and breastfeeding [5, 11]. The projected increase in mortality rates, especially among women, harkens back to historical events such as the 1984/85 Ethiopian famine, where women, especially the young, suffered disproportionately due to unequal access to resources [12].

Changing weather patterns and rising temperatures elevate the risk of malaria transmission, with about 55% of the world's malaria cases found in the 33 nations comprising Central Africa, South Africa, East Africa, and West Africa [6]. Prenatal malaria infections in these regions contribute to underweight births and maternal severe illness, leading to an estimated 50% mortality rate [13]. Increased temperatures and food insecurity further contribute to health risks, including fatigue, hypertension, stillbirths, and miscarriages [14].

In rural regions without access to renewable energy, women experience greater respiratory diseases, often resorting to using dangerous conventional fuels for cooking, resulting in exposure to poisonous contaminants [15]. The increased nutritional requirements during menstruation and childbirth contribute to higher rates of anemia among women globally [7]. Alarmingly, a UNICEF report from 2022 highlighted a doubling of female child marriages on average in just one year in drought‐affected regions [16]. In Ethiopia, underage marriages increased by an average of 119% in these areas, accompanied by coercive practices such as female genital mutilation and an upsurge in violence against women and girls [16].

Climate change–induced droughts have led women and girls to walk up to 30 km, more than three times the previous distance, in search of water and other necessities in Kenya. This increased vulnerability exposes them to heightened instances of gender‐based violence, domestic violence, and sexual assault, as evidenced by reports from Somaliland in January 2022, where more than 50% of cases occurred in certain areas [16, 17]. The implications of climate change not only persist but also widen gender gaps in income, education, health, and work involvement among women. This perpetuates ongoing socio‐economic and structural circumstances, further escalating tensions above the conflict and violence threshold [6].

The intersection of racial and gender inequality with climate change is evolving into the primary cause of significant injustices. The systematic undervaluation and exploitation of black women within capitalist systems, the disempowerment of marginalized communities, the lack of representation of women and racial minorities in climate decision‐making, biases in portrayals, and the disregard for historical legacies of racism and sexism are just a few examples of these injustices [18]. In addition, disasters affect much more women, forcing them into displacement in precarious conditions and exposing them to violences and exploitations [19, 20]. For instance, migrating rural women and girls face the heightened risk of being trafficked for sexual exploitation at a prevalence of 78%, working in hazardous jobs, and encountering racism, xenophobia, and various forms of discrimination in East Africa [21].

## RECOMMENDATIONS

To effectively address the profound impact of climate change on women in Africa, there is a critical need to adopt integrated policies and programs that go beyond conventional strategies. Recognizing the pivotal role women play in combating climate change, the strategy of gender mainstreaming emphasizes effective communication and interaction with women across all tiers and sectors. This approach advocates for a nuanced and targeted response, acknowledging the unique challenges faced by women in the context of climate change and seamlessly integrating gender considerations into broader climate‐related initiatives and goals. Policymakers and stakeholders are urged to conduct systematic analyses of gender dynamics, set gender‐sensitive benchmarks, and fully utilize the leadership roles of women in disaster management and adaptation.

Multisectoral approaches are essential for addressing the diverse impacts of climate change on women's health. The significance of research, education, and heightened sectoral coordination emerge as essential elements of a holistic strategy. Policymakers and other stakeholders should invest in detailed studies at the household level to understand the dynamics of relationships and decision‐making processes. Examining how gender interacts with socio‐economic factors provides insights into the unique challenges faced by women in their daily lives. Education, training, and upskilling opportunities are crucial for raising awareness about climate change, promoting participation in climate‐related programs, and offering tailored adaptation measures.

The approach also places a strong emphasis on closing gender gaps in asset ownership and empowerment. Climate action should advocate for the development and enforcement of laws and policies that not only recognize gender differences but also safeguard women's rights, especially regarding property ownership, with a focus on land rights. Gender‐responsive solutions are designed to reduce existing gender disparities and promote equality. Women should be equipped with training in new technologies and rights, including land ownership and production. Access to productive resources, financial capital, essential services like healthcare and childcare, and streamlined processes for formalizing enterprises are crucial components of the recommended framework. Facilitating access to markets, land, labor, finance, and products enables women to assert agency and economic independence in the face of climate change challenges.

## CONCLUSION

The overarching narrative of developmental theory emphasizes the significance of empowering women to take control of their lives, particularly in the face of climate change. This all‐encompassing strategy strives to increase the power of women and lessen their vulnerability to climate‐related dangers. Until such a paradigm shift occurs, the disastrous implications of climate change and gender inequalities on women will persist, hindering sustainable development in Africa. The call for integrated strategies and comprehensive action is paramount, recognizing that climate change is not merely an environmental issue but a complex web of social, economic, and political challenges.

## AUTHOR CONTRIBUTIONS

Olalekan John Okesanya: Khlood Fathi Hassan Alnaeem: Hakeem Kayode Hassan: Adebimpe Tolutope Oso: Olaniyi Abideen Adigun: Oumnia Bouaddi: Noah Olabode Olaleke: Sara Gabrallah M.kheir: Usman Abubakar Haruna: Deborah Oluwaseun Shomuyiwa: Emery Manirambona: and Melat Tesfaye Asebot: Conceived and designed the research; analyzed; performed the research; wrote the paper; proofread the paper; review‐editing; and wrote the final draft.

## CONFLICT OF INTEREST STATEMENT

The authors declare no conflicts of interest. Deborah Oluwaseun Shomuyiwa, Emery Manirambona, Oumnia Bouaddi, and Usman Abubakar Haruna are Editorial Board members of Public Health Challenges and co‐authors of this article. To minimize bias, they were excluded from all editorial decision‐making related to the acceptance of this article for publication.

## FUNDING INFORMATION

No funding was received for this work.

## ETHICS STATEMENT

No ethical approval was required for this article.

## Data Availability

The data that support the findings of this study are available from the corresponding author upon reasonable request
